# Bakuchiol Alleviates Hyperglycemia-Induced Diabetic Cardiomyopathy by Reducing Myocardial Oxidative Stress via Activating the SIRT1/Nrf2 Signaling Pathway

**DOI:** 10.1155/2020/3732718

**Published:** 2020-09-30

**Authors:** Wenshuai Ma, Wangang Guo, Fujun Shang, Yan Li, Wei Li, Jing Liu, Chao Ma, Jiwei Teng

**Affiliations:** Department of Cardiology, Second Affiliated Hospital, The Air Force Medical University, 1 Xinsi Road, Xi'an 710038, China

## Abstract

Bakuchiol (BAK), a monoterpene phenol reported to have exerted a variety of pharmacological effects, has been related to multiple diseases, including myocardial ischemia reperfusion injury, pressure overload-induced cardiac hypertrophy, diabetes, liver fibrosis, and cancer. However, the effects of BAK on hyperglycemia-caused diabetic cardiomyopathy and its underlying mechanisms remain unclear. In this study, streptozotocin-induced mouse model and high-glucose-treated cell model were conducted to investigate the protective roles of BAK on diabetic cardiomyopathy, in either the presence or absence of SIRT1-specific inhibitor EX527, SIRT1 siRNA, or Nrf2 siRNA. Our data demonstrated for the first time that BAK could significantly abate diabetic cardiomyopathy by alleviating the cardiac dysfunction, ameliorating the myocardial fibrosis, mitigating the cardiac hypertrophy, and reducing the cardiomyocyte apoptosis. Furthermore, BAK achieved its antifibrotic and antihypertrophic actions by inhibiting the TGF-*β*1/Smad3 pathway, as well as decreasing the expressions of fibrosis- and hypertrophy-related markers. Intriguingly, these above effects of BAK were largely attributed to the remarkable activation of SIRT1/Nrf2 signaling, which eventually strengthened cardiac antioxidative capacity by elevating the antioxidant production and reducing the reactive oxygen species generation. However, all the beneficial results were markedly abolished with the administration of EX527, SIRT1 siRNA, or Nrf2 siRNA. In summary, these novel findings indicate that BAK exhibits its therapeutic properties against hyperglycemia-caused diabetic cardiomyopathy by attenuating myocardial oxidative damage via activating the SIRT1/Nrf2 signaling.

## 1. Introduction

Diabetes mellitus (DM), a chronic endocrine metabolic disorder characterized by long-standing hyperglycemia, has become more prevalent in recent years and seriously threatens the human health [1, 2]. In people with DM, the existence of abnormal myocardial structure and performance has led to the descriptive terminology diabetic cardiomyopathy [3]. These diabetes-associated changes, featured by the presence of myocardial fibrosis, pathological remodeling, and associated cardiac dysfunction, occur independent of conventional cardiovascular risk factors, such as coronary artery disease, valvular disease, hypertension, and dyslipidemia [4, 5]. Until now, no efficacious approaches have been applicated in clinical trials that can definitely alleviate this specific form of heart disease. Therefore, it is of great significance to search for a therapeutic substance for treating diabetic cardiomyopathy.

Oxidative stress, induced by glycometabolism disorder, is widely accepted to be one of the key pathophysiological mechanisms in the development of diabetic cardiomyopathy [6, 7]. Simultaneously, under the condition of prolonged hyperglycemia, reactive oxygen species (ROS) are excessively produced in the myocardium due to impaired mitochondrial oxidative phosphorylation which, in turn, intensifies myocardial oxidative stress, aggravates mitochondrial dysfunction, and accelerates the progression of myocardial fibrosis and hypertrophy [8–10]. Thus, finding agents that alleviate oxidative stress and inhibit ROS generation may represent as a practicable therapy for diabetic cardiomyopathy.

Bakuchiol (BAK), a bioactive monoterpene phenol, can be vastly extracted from the seeds of *Psoralea corylifolia*. Previous literatures have demonstrated that BAK exerts extensive pharmacological properties, including antioxidant, antibacterial, anti-inflammatory, antiaging, and estrogen-like effects [11, 12]. Given its wide spectrum of biological activities, especially the antioxidative capability, BAK has been verified to exhibit protective roles against multiple diseases in various organs or tissues [13–16]. Additionally, experimental evidence revealed that BAK also possessed promising potency on substance metabolism in diabetic animal models [17, 18]. However, whether BAK treatment could prevent the occurrence and progression of cardiomyopathy in diabetic hearts and the underlying mechanisms remain ambiguous.

Silent information regulator 1 (SIRT1), one of the sirtuin family distinguished by holding a highly conserved nicotinamide adenine dinucleotide- (NAD^+^-) binding catalytic domain, is deemed to be responsible for the beneficial effects in the development and treatment of cardiovascular diseases [19, 20]. Intriguingly, in recent years, SIRT1 has attracted widespread attentions as a protein modulator and stress adaptor related to hyperglycemia attenuating cardiac dysfunction and improving diabetic cardiomyopathy [21, 22]. Moreover, nuclear factor-erythroid 2-related factor 2 (Nrf2), an important transcription factor that binds to antioxidant-responsive elements (AREs), shows an outstanding performance in attenuating oxidative damage to consolidate the cellular defense system against diabetic cardiomyopathy [23, 24]. Numerous studies have also clearly indicated that SIRT1 could manifest its antioxidative effects via the activation of Nrf2 [25, 26]. Therefore, it is of great interest to probe whether SIRT1/Nrf2 signaling plays a vital role in the amelioration actions of BAK against diabetic cardiomyopathy.

Herein, this study was carried out aimed at exploring the pharmacological effects of BAK in the setting of hyperglycemia-induced diabetic cardiomyopathy and elucidate whether SIRT1/Nrf2 signaling is involved in its protective molecular mechanisms both *in vivo* and *in vitro*.

## 2. Materials and Methods

### 2.1. Reagents

BAK (purity ≥ 98%) was purchased from Winherb Medical Co. (Shanghai, China). Streptozotocin (STZ) and EX527 were purchased from Sigma-Aldrich (St. Louis, MO, USA). The *In Situ* Cell Death Detection Kit was purchased from Roche Biochemicals (Mannheim, Germany) to detect the cell apoptosis. Kits for determining glutathione peroxidase (GSH-Px), superoxide dismutase (SOD) activities, and malondialdehyde (MDA) content were obtained from Nanjing Jiancheng Bioengineering Institute (Nanjing, China). The fluorescent probe 2′,7′-dichlorofluorescein diacetate (DCFH-DA) was purchased from Beyotime Institute of Biotechnology (Shanghai, China) to detect intracellular ROS generation. Dihydroethidium (DHE) was purchased from Invitrogen (Carlsbad, CA, USA) for detecting ROS generation in the myocardium. Primary antibodies against p-Smad3, t-Smad3, and *β*-actin were purchased from Cell Signaling Technology (Boston, MA, USA). Primary antibodies against SIRT1, Nrf2, transforming growth factor-*β1* (TGF-*β1*), *α*-smooth muscle actin (*α*-SMA), and Histone H3 were purchased from Abcam (Cambridge, MA, USA). The rabbit anti-goat and goat anti-mouse secondary antibodies were obtained from Zhongshan Company (Beijing, China).

### 2.2. Animals and In Vivo Protocols

Healthy C57BL/6 male mice (weighing 20-25 g at the age of 8 weeks) were obtained from the Experimental Animal Center of the Air Force Medical University. Animals were housed at 22-24°C under a specific pathogen-free environment with a 12/12 h light/dark cycle and had free access to food and water. All of the animal care and experimental procedures conformed to the Guidelines for the Care and Use of Laboratory Animals published by the United States National Institutes of Health (NIH Publication, revised 2011). All procedures were reviewed and approved by the Air Force Medical University Committee on Animal Care.

After being fed with high-fat diet (HFD) for 4 weeks, diabetes was induced by intraperitoneal injection of STZ (dissolved in 0.1 mM sodium citrate (pH 4.5), 60 mg/kg) for 3 consecutive days. The age-matched control mice received the same volume of vehicle injection (sodium citrate). Two weeks after the final STZ injection, mice with fasting blood glucose above 11.1 mM were defined as diabetic, which were continued to be fed with HFD in the following experiments. Animals were randomly divided into 4 groups (*n* = 15): (1) control group, (2) diabetic mouse (DM) group, (3) DM+BAK group, and (4) DM+BAK+EX527 group. Groups (3) and (4) were orally administrated with BAK (60 mg/kg/d, first dissolved in ethanol and then diluted in sterile saline) for 12 weeks, while groups (1) and (2) received the same volume of vehicle alone. EX527 (5 mg/kg/d, first dissolved in ethanol and then diluted in sterile saline) was intraperitoneally administered twice a week for 12 weeks.

### 2.3. Cell Culture and Treatment

H9c2 cardiomyoblast cell line was purchased from the American Type Culture Collection (ATCC). Cells were maintained in Dulbecco's modified Eagle's medium (DMEM, Gibco, Grand Island, NY, USA), supplemented with 10% heat-inactivated bovine calf serum (FBS, Gibco, Grand Island, NY, USA) and 1% Penicillin-Streptomycin Solution at 37°C with 5% CO_2_.

In the high-glucose (HG) group, cells were cultured in DMEM containing 33 mM D-glucose to mimic the plasma level of glucose from diabetic mice, while cells in the control group were incubated in normal glucose medium containing 5.5 mM glucose, supplemented with 27.5 mM mannitol to achieve the osmotic pressure at 33 mM. At the same time, cells in the BAK-treated groups were cultured in a high-glucose medium with additional 2, 5, and 10 *μ*M of BAK.

### 2.4. Echocardiography

As described previously [27], transthoracic echocardiography was performed with a VisualSonics Vevo 770 ultrasound system (Toronto, Ontario, Canada) to evaluate cardiac function. Animals were anesthetized with 1.0% isoflurane and placed on a thermostabilized pad, while the heart rates should be maintained at 400-500 beats per minute during the whole process. Motion- (M-) mode echocardiographic images were recorded at the level of the papillary muscles to calculate the left ventricular end-systolic volumes (LVESV), left ventricular end-diastolic volumes (LVEDV), left ventricular ejection fraction (LVEF), and left ventricular fractional shortening (LVFS).

### 2.5. HMI, LVMI, and HW/TL Assessment

After the body weight was measured, the animals were euthanized, and their hearts and right lower extremity tibias were immediately harvested and rinsed with phosphate-buffered saline (PBS). After being placed on filter paper to blot moisture, the total heart mass, left ventricular mass, and tibial length of each mouse were measured. The total heart mass index (HMI, total heart mass/body mass), left ventricular mass index (LVMI, left ventricular mass/body mass), and heart weight/tibial length (HW/TL) ratio were calculated to evaluate the cardiac hypertrophy.

### 2.6. Histological and Immunohistochemistry Staining

Tissues were fixed in 4% paraformaldehyde solution, embedded in paraffin, and sectioned at 5 *μ*m thickness. Hematoxylin-eosin (HE), wheat germ agglutinin (WGA), or Masson trichrome staining was used to examine the cross-sectional area of cardiomyocyte and extracellular collagen deposition, respectively. Six random fields in each sample were captured to assess the quantitative difference in the cross-sectional area and collagen content with the ImageJ software (NIH, Bethesda, MD, USA). For immunohistochemistry staining, heart sections were deparaffinized in xylene and rehydrated. After antigen retrieval was achieved with citrate (pH = 6), the slides were washed in PBS. Primary antibodies (collagen І (1 : 50) and collagen III (1 : 50)) were applied overnight at 4°C in PBS containing 2% bovine serum albumin (BSA). The sections were then washed in PBS followed by incubation with anti-mouse or rabbit secondary antibodies labeled with horse radish peroxidase. Staining was achieved by completing the DAB (3,3′-diaminobenzidine tetrahydrochloride) and hematoxylin counterstain process. All images were obtained by the bright field microscope (Olympus CX41, Olympus, Tokyo, Japan).

### 2.7. Cell Viability

Cell counting kit (CCK-8 kit, Dojindo, Kumamoto, Japan) was used to evaluate cell viability. Procedures were carried out according to the manufacturer's protocol. Cells of different groups were counted, adjusted the density to 1 × 10^5^ cells per well, and seeded in a 96-well plate. CCK-8 (10 *μ*L) solution was added in each well, and the plate was incubated darkly for 2 h. The absorbance at 450 nm was detected using a microplate reader. All data were normalized to the control well, which contained no cells but normal culture media.

### 2.8. Assessment of SOD Activity, GSH-Px Activity, and MDA Content

The activities for SOD and GSH-Px and content of MDA in heart tissues and cultured cells were analyzed spectrophotometrically with a SpectraMax M5 device, following the instructions of the special commercial kits.

### 2.9. TUNEL Assay

Cell apoptosis was analyzed via TUNEL staining using the *In Situ* Cell Death Detection Kit according to the manufacturer's protocol. All the cells exhibited blue nuclear after DAPI (4′,6-diamidino-2-phenylindole) staining, while the TUNEL-positive cells exhibited green nuclear staining. Five randomly selected fields were observed using an Olympus FV10C-W3 laser confocal microscope (Olympus, Tokyo, Japan). The apoptotic index was calculated to reflect the degree of apoptosis and expressed as the number of apoptotic positive cells/total cells.

### 2.10. ROS Detection

As previously described [28], the frozen tissue sections were stained with DHE reaction mixture to detect ROS generation in the myocardium. Each sample was examined with an Olympus FV10C-W3 laser confocal microscope, and the fluorescent intensity in different groups was determined by the ImageJ software. Intracellular ROS production was measured after H9c2 cells in each group were treated with DCFH-DA reaction mixture [29]. A microplate reader (SpectraMax M5) was used to measure fluorescent intensity at an emission of 535 nm (excitation at 485 nm). The amount of emitted fluorescence was correlated with the quantity of ROS volume.

### 2.11. siRNA Transfection

To knock down the SIRT1 and Nrf2 expressions, predesigned and validated siRNAs along with corresponding negative control (GenePharma, Suzhou, China) were transfected into H9c2 cells for 24 h by Lipofectamine™ 3000 transfection reagent (Invitrogen, Carlsbad, CA, USA). The sense and antisense sequences of SIRT1 siRNA and Nrf2 siRNA are listed in Table 1, and the siRNAs were transiently transfected into H9c2 cells with Lipofectamine 3000 according to the manufacturer's instructions for 24 h in OPTI-MEM medium (Gibco, Carlsbad, CA, USA).

### 2.12. RT-PCR and Western Blot

As previously described [30, 31], real-time polymerase chain reaction (RT-PCR) was performed with the CFX96 system-C1000 Thermal Cycler (Bio-Rad Laboratories, Hercules, CA, USA) and western blotting was analyzed by Image Lab 5.2.1 (Bio-Rad Laboratories, Hercules, CA, USA). The primer sequences used are listed in Table 2, and the concentrations of antibodies are SIRT1 (1 : 1000), Nrf2 (1 : 1000), TGF-*β1* (1 : 1000), *α*-SMA (1 : 1000), p-Smad3 (1 : 1000), t-Smad3 (1 : 1000), H3 (1 : 1000), and *β*-actin (1 : 5000).

### 2.13. Statistical Analysis

Data were presented as mean ± standard error of the mean (SEM). The statistical significance of differences was determined using two-tailed Student's *t* test and one-way ANOVA, followed by a Bonferroni multiple-comparison test, using GraphPad Prism 5 (GraphPad Software, San Diego, CA, USA). *P* < 0.05 was considered to indicate significant differences.

## 3. Results

### 3.1. BAK Upregulated the Expressions of SIRT1 and Nrf2 in Diabetic Myocardium

**Figure 1:**
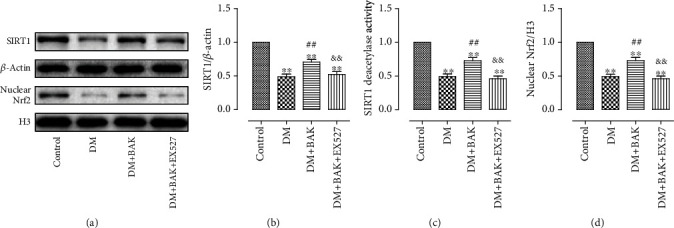
BAK treatment increased the expression and activity of SIRT1 and nuclear accumulation of Nrf2 in diabetic hearts. (a) Representative blots. (b) SIRT1 expression. (c) Relative SIRT1 activity. (d) Nrf2 nuclear translocation. Data are presented as the mean ± SEM (*n* = 6 in each group). ^∗/∗∗^*P* < 0.05/0.01 vs. the control group, ^#/##^*P* < 0.05/0.01 vs. the DM group, and ^&/&&^*P* < 0.05/0.01 vs. the DM+BAK group.

To explore the protective roles of BAK on hyperglycemia-induced cardiac damages in diabetic mice, as well as the underlying molecular mechanisms, SIRT1-specific inhibitor EX527 was employed in our *in vivo* experiments. As shown in Figure 1, the expression and activity of SIRT1 were notably decreased in the diabetic myocardium compared with the control group, accompanied by the prominently reduced nuclear translocation of Nrf2, while treatment with BAK markedly restored SIRT1 expression and deacetylation activity and Nrf2 nuclear accumulation. Simultaneously, as exhibited in Figure S1, BAK alone could not increase the expression and deacetylation activity of SIRT1 or promote the nuclear accumulation of Nrf2 under a normal condition, indicating that the upregulation of SIRT1 and Nrf2 is a secondary outcome of the BAK treatment in a pathological state. Taken together, the results suggested that SIRT1 and Nrf2 signaling might mediate the cardioprotective action of BAK in diabetic hearts.

### 3.2. EX527 Abolished BAK-Induced Alleviation of Myocardial Dysfunction in Diabetic Mice

**Figure 2:**
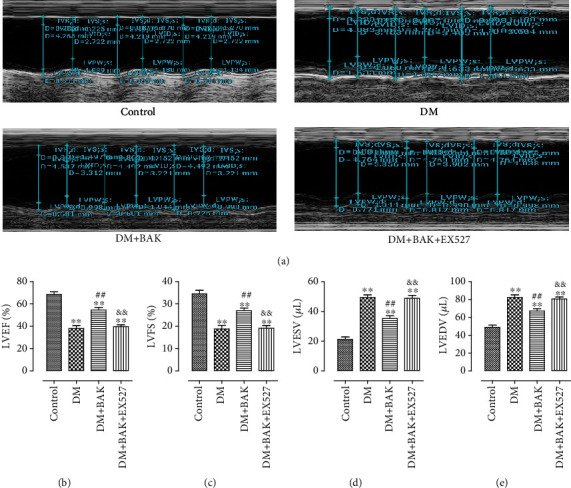
EX527 abolished BAK-induced cardioprotective effects in diabetic mice. (a) Representative M-mode images by echocardiography. (b) Left ventricular ejection fraction (LVEF). (c) Left ventricular fractional shortening (LVFS). (d) Left ventricular end-systolic volume (LVESV). (e) Left ventricular end-diastolic volume (LVEDV). Data are presented as the mean ± SEM (*n* = 6 in each group). ^∗/∗∗^*P* < 0.05/0.01 vs. the control group, ^#/##^*P* < 0.05/0.01 vs. the DM group, and ^&/&&^*P* < 0.05/0.01 vs. the DM+BAK group.

As echocardiography recorded in Figure 2(a), cardiac functions were markedly impaired in the DM group, as confirmed by decreased LVEF and LVFS, while BAK treatment significantly increased both LVEF and LVFS in the DM+BAK group. Moreover, cardiac dysfunction was markedly alleviated with BAK supplementation, as evidenced by reduced LVESV and LVEDV compared with the DM group (Figures 2(d) and 2(e)). Nevertheless, these cardioprotective activities were all blunted by EX527 administration, suggesting that SIRT1 signaling played a vital role in the amelioration effects of BAK on cardiac dysfunction in diabetic mice.

### 3.3. EX527 Blocked BAK-Induced Mitigation of Cardiac Hypertrophy in Diabetic Mice

**Figure 3:**
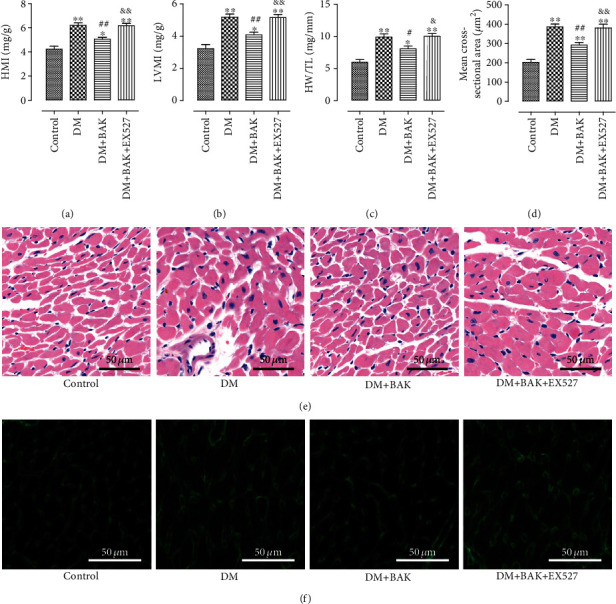
EX527 blocked BAK-induced mitigation of cardiac hypertrophy in diabetic mice. (a) Heart mass index (HMI). (b) Left ventricular mass index (LVMI). (c) Heart weight/tibia length (HW/TL) ratio. (d) Mean cross-sectional area of individual cardiomyocyte. (e) Representative images of HE staining of myocardial tissue (scale bar = 50 *μ*m). (f) Representative images of WGA staining of myocardial tissue (scale bar = 50 *μ*m). Data are presented as the mean ± SEM (*n* = 6 in each group). ^∗/∗∗^*P* < 0.05/0.01 vs. the control group, ^#/##^*P* < 0.05/0.01 vs. the DM group, and ^&/&&^*P* < 0.05/0.01 vs. the DM+BAK group.

Cardiac hypertrophy is a prominent characteristic of diabetic hearts induced by prolonged hyperglycemia. Consistent with the findings from other papers [6, 32], mice in the DM group showed remarkable increased HMI, LVMI, and enhanced HW/TL ratio (Figures 3(a)–3(c)), while BAK treatment markedly mitigated these changes, as evidenced by the obviously decreased HMI and LVMI and declined HW/TL ratio. However, EX527 administration reversed the favorable ameliorative effects of BAK. As shown in Figures 3(e) and 3(f), HE staining and WGA staining of the cross-sections of the left ventricle are more intuitive ways to assess the cardiac hypertrophic response in each group. Compared with the DM group, BAK treatment greatly reduced the mean cross-sectional area of the left ventricular myocytes. Consistently, EX527 administration abolished the outstanding curative effect of BAK on delaying the progression of cardiac hypertrophy (Figure 3(d)).

**Figure 4:**
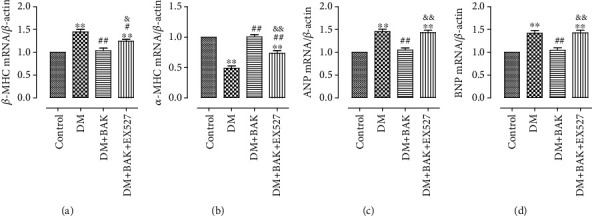
EX527 blunted BAK-induced suppression on activation of hypertrophy-related genes in diabetic myocardium. (a) *β*-MHC mRNA level. (b) *α*-MHC mRNA level. (c) ANP mRNA level (8W). (d) BNP mRNA level. Data are presented as the mean ± SEM (*n* = 6 in each group). ^∗/∗∗^*P* < 0.05/0.01 vs. the control group, ^#/##^*P* < 0.05/0.01 vs. the DM group, and ^&/&&^*P* < 0.05/0.01 vs. the DM+BAK group.

Myosin heavy chain (MHC) is the basic unit of myosin and plays an important role in ensuring the normal function of muscle cells. The two isoforms of myosin heavy chain, *α*-MHC and *β*-MHC, will undergo canonical MHC changes from *α*-MHC to *β*-MHC in the hypertrophic hearts [33]. In accordance with previous studies [6, 34], *α*-MHC was downregulated and *β*-MHC was upregulated in mRNA levels in the hearts of the DM group, while BAK administration reversed this transformation in MHC expression (Figures 4(a) and 4(b)). Moreover, the accurate biomarkers for myocardial expansion-caused cardiac insufficiency, ANP and BNP, were markedly raised in mRNA levels in the DM group as well. Similarly, BAK treatment inhibited the alteration (Figures 4(d) and 4(e)). However, the above BAK-induced suppression on activation of hypertrophy-related genes was significantly invalidated by EX527.

These data indicated that BAK mitigated cardiac hypertrophy in diabetic mice in a SIRT1-dependent manner.

### 3.4. EX527 Blunted BAK-Induced Amelioration of Myocardial Fibrosis and Collagen Deposition in Diabetic Myocardium

**Figure 5:**
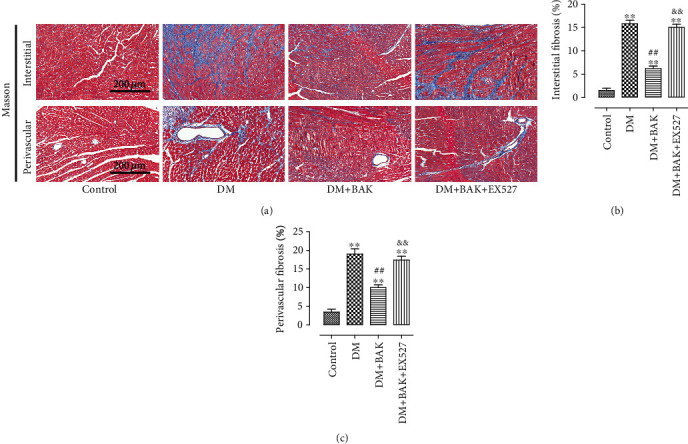
EX527 inhibited BAK-induced amelioration of myocardial fibrosis in diabetic myocardium. (a) Representative images of Masson's trichrome staining of the cardiac interstitial and perivascular areas (scale bar = 200 *μ*m). (b) Mean cardiac interstitial fibrosis. (c) Mean cardiac perivascular fibrosis. Data are presented as the mean ± SEM (*n* = 6 in each group). ^∗/∗∗^*P* < 0.05/0.01 vs. the control group, ^#/##^*P* < 0.05/0.01 vs. the DM group, and ^&/&&^*P* < 0.05/0.01 vs. the DM+BAK group.

Cardiac fibrosis is a critical hallmark in the process of cardiac remodeling caused by diabetic cardiomyopathy, which further contributes to worsening of the already compromised cardiac functions [35, 36]. To investigate the antifibrotic properties of BAK treatment in diabetic hearts, we detected the level of collagen deposition in myocardium tissue by Masson staining. Representative images of Masson staining for the interstitial and perivascular areas are shown in Figure 5(a), respectively. Markedly, compared to the DM group, BAK administration significantly attenuated collagen accumulation both in the interstitial and perivascular areas in the DM+BAK group (Figures 5(b) and 5(c)). Of note, these antifibrotic effects of BAK were markedly reversed by EX527.

**Figure 6:**
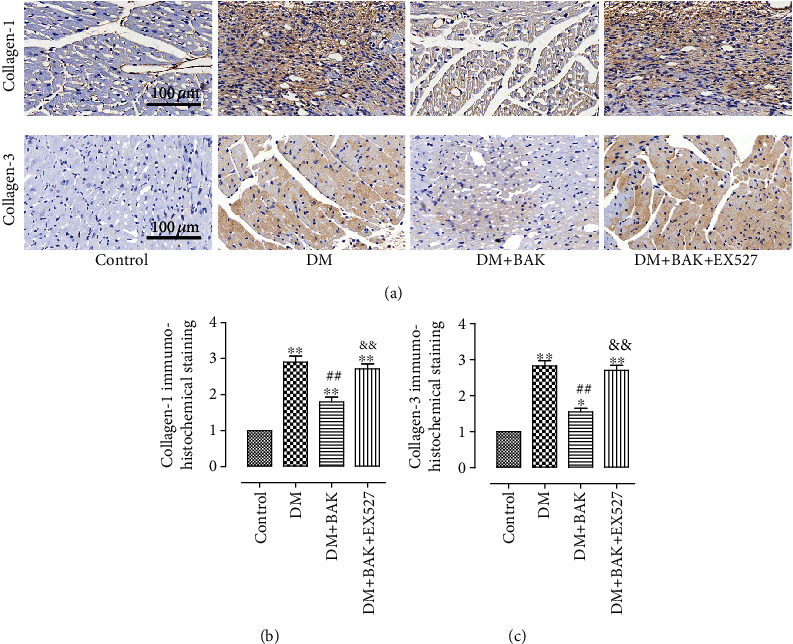
EX527 blunted BAK-induced amelioration of collagen deposition in diabetic myocardium. (a) Representative images of immunohistochemistry of collagen І/III (scale bar = 100 *μ*m). (b) Collagen І immunohistochemical expression. (c) Collagen III immunohistochemical expression. Data are presented as the mean ± SEM (*n* = 6 in each group). ^∗/∗∗^*P* < 0.05/0.01 vs. the control group, ^#/##^*P* < 0.05/0.01 vs. the DM group, and ^&/&&^*P* < 0.05/0.01 vs. the DM+BAK group.

Immunohistochemical staining of collagen І and collagen III was also conducted to further determine the alleviative effect of BAK treatment on cardiac fibrosis. A similar trend consistent with Masson staining is shown in Figure 6, revealing that BAK treatment could restrain the deposition of collagen І and collagen III, compared to the DM group. Intriguingly, the amelioration of BAK on this pathologic characteristic was blunted with the administration of EX527 as well.

Collectively, these data demonstrated that BAK displayed its protective properties against myocardial fibrosis and collagen deposition in diabetic myocardium via SIRT1 signaling.

### 3.5. EX527 Counteracted BAK-Induced Inhibition of Synthesis of Fibrosis-Associated Proteins in Diabetic Myocardium

**Figure 7:**
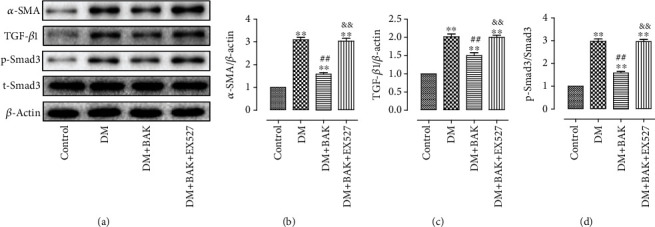
EX527 attenuated BAK-induced restrained stimulation of the TGF-*β1*/Smad3 pathway in diabetic myocardium. (a) Representative blots. (b) *α*-SMA expression. (c) TGF-*β1* expression. (d) p-Smad3 expression. Data are presented as the mean ± SEM (*n* = 6 in each group). ^∗/∗∗^*P* < 0.05/0.01 vs. the control group, ^#/##^*P* < 0.05/0.01 vs. the DM group, and ^&/&&^*P* < 0.05/0.01 vs. the DM+BAK group.

As shown in Figures 7(a) and 7(b), the antifibrotic effectiveness of BAK was further verified by inhibiting the expression level of the cardiac fibrotic marker *α*-SMA, which was dramatically elevated in the DM group and could be significantly reversed by EX527 treatment. Moreover, the TGF-*β1*/Smad3 signaling pathway, playing a key role in mediating ROS generation to pathologic fibrosis, was further measured in our research. As shown in Figures 7(a), 7(c), and 7(d), the TGF-*β1*/Smad3 pathway was evidently inhibited after BAK treatment compared with the DM group. Consistently, this phenomenon was prominently blunted by EX527 administration, indicating that expressive suppression of the TGF-*β1*/Smad3 signaling was involved in the noteworthy benefit of BAK to alleviate cardiac fibrosis in diabetic hearts via SIRT1-induced inhibition of ROS generation.

### 3.6. EX527 Attenuated BAK-Induced Suppression of the Oxidative Stress in Diabetic Myocardium

**Figure 8:**
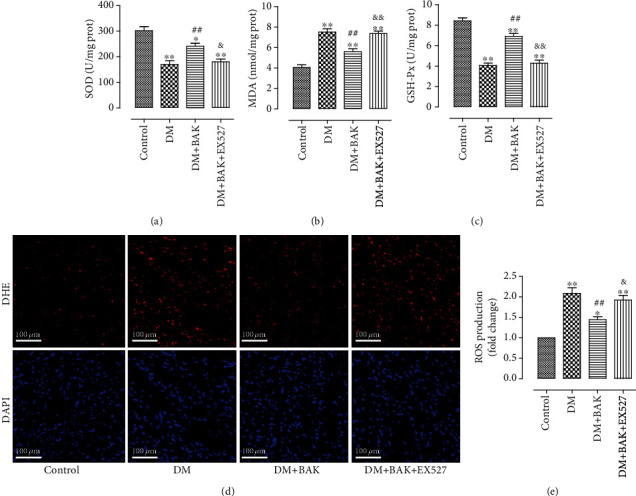
EX527 blunted BAK-induced suppression on oxidative stress level in diabetic myocardium. (a) Myocardial SOD activity. (b) Myocardial MDA contents. (c) Myocardial GSH-Px activity. (d) Representative images of DHE staining (scale bar = 100 *μ*m). (e) DHE intensity. Data are presented as the mean ± SEM (*n* = 6 in each group). ^∗/∗∗^*P* < 0.05/0.01 vs. the control group, ^#/##^*P* < 0.05/0.01 vs. the DM group, and ^&/&&^*P* < 0.05/0.01 vs. the DM+BAK group.

As shown in Figures 8(a)–8(c), BAK exhibited its antioxidative capabilities by markedly increasing the enzymatic activities of SOD and GSH-Px in myocardial tissues in the DM+BAK group, together with observably decreased MDA content. However, all these effects were significantly reversed by EX527 administration. Furthermore, compared with the DM group, BAK also verified its antioxidant capacity by inhibiting ROS production in the diabetic hearts, which was shown by the declined intensity of DHE fluorescence (Figures 8(d) and 8(e)). Nonetheless, EX527 administration dramatically abolished this effect. Those results confirmed that SIRT1 signaling participated in the ameliorative effects of BAK against myocardial oxidative stress *in vivo.*

### 3.7. BAK Markedly Enhanced the Expressions of SIRT1 and Nrf2 in HG-Treated H9c2 Cells

**Figure 9:**
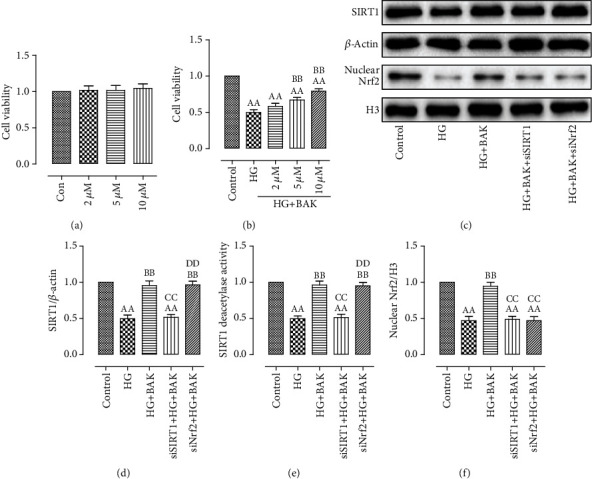
BAK treatment increased cell viability, raised intracellular SIRT1 expression and activity, and enhanced Nrf2 nuclear accumulation in HG-treated H9c2 cells. (a) BAK treatment (2 *μ*M, 5 *μ*M, and 10 *μ*M) had no toxic effect on the cell viability of control H9c2 cells. (b) BAK treatment dose dependently increased the cell viability in HG-treated H9c2 cells. (c) Representative blots. (d) SIRT1 expression. (e) Relative SIRT1 activity. (f) Nrf2 nuclear translocation. Data are presented as the mean ± SEM (*n* = 6 in each group). ^aa^*P* < 0.01 vs. the control group, ^bb^*P* < 0.01 vs. the HG group, ^cc^*P* < 0.01 vs. the HG+BAK group, and ^dd^*P* < 0.01 vs. the siSIRT1+HG+BAK group.

To further verify the positive effects of BAK against HG-induced damages on cardiomyocytes and illuminate the molecular mechanisms, we used cardiomyoblast-derived H9c2 cells and employed the SIRT1 siRNA and Nrf2 siRNA to conduct the *in vitro* experiments. As shown in Figures 9(a) and 9(b), we first investigated that BAK treatment alone had no toxic effect on cell survival when H9c2 cells were incubated with normal glucose, while it could significantly increase cell viability following HG treatment dose dependently. This protective effect of BAK was most obvious at the concentration of 10 *μ*M, which was selected for further studies.

Next, as results exhibited in Figures 9(c)–9(f), compared with the control group, we also observed that the expression and activity of SIRT1 were significantly decreased after HG incubation, along with the markedly decreased translocation of nuclear Nrf2. Simultaneously, treatment with BAK prominently restored SIRT1 expression and deacetylation activity and increased Nrf2 nuclear accumulation, which further indicated that BAK might exert its protective capabilities by upregulating the SIRT1 and Nrf2 signaling *in vitro*. Additionally, in accordance with our *in vivo* data, the results in Figure S2 confirmed that the changes of intracellular SIRT1 expression and activity and Nrf2 nuclear accumulation were a secondary outcome of the BAK treatment as well.

### 3.8. SIRT1 siRNA and Nrf2 siRNA Abolished BAK-Induced Prevention on Cardiomyocyte Death and Oxidative Stress in HG-Treated H9c2 Cells

**Figure 10:**
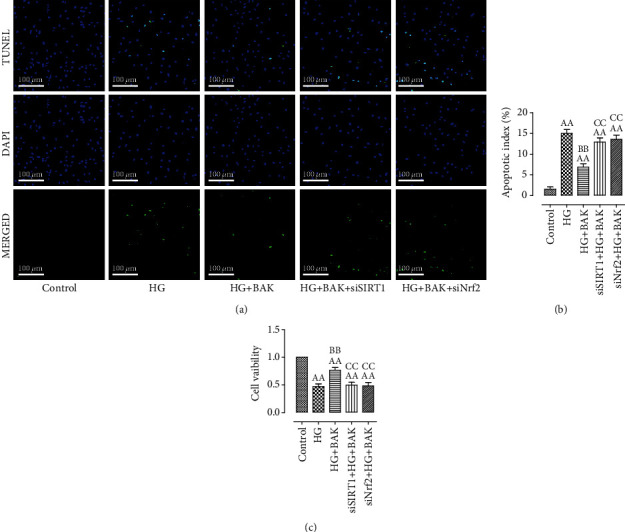
SIRT1 siRNA and Nrf2 siRNA transfection blunted BAK-induced antiapoptotic effect in HG-treated H9c2 cells. (a) Representative images of TUNEL staining (scale bar = 100 *μ*m). The apoptotic cells were detected by TUNEL (green), and the nuclei were detected by DAPI (blue). (b) Cellular apoptotic index. (c) Cell viability. Data are presented as the mean ± SEM (*n* = 6 in each group). ^aa^*P* < 0.01 vs. the control group, ^bb^*P* < 0.01 vs. the HG group, and ^cc^*P* < 0.01 vs. the HG+BAK group.

As shown in Figures 10(a) and 10(b), BAK significantly prevented HG-induced cardiomyocyte apoptosis by decreasing TUNEL-positive nuclei and apoptotic ratio compared with the HG group. Moreover, cell viability in the HG+BAK group was markedly elevated with BAK supplementation (Figure 10(c)). However, these beneficial effects of BAK were notably blunted by SIRT1 siRNA and Nrf2 siRNA administration.

**Figure 11:**
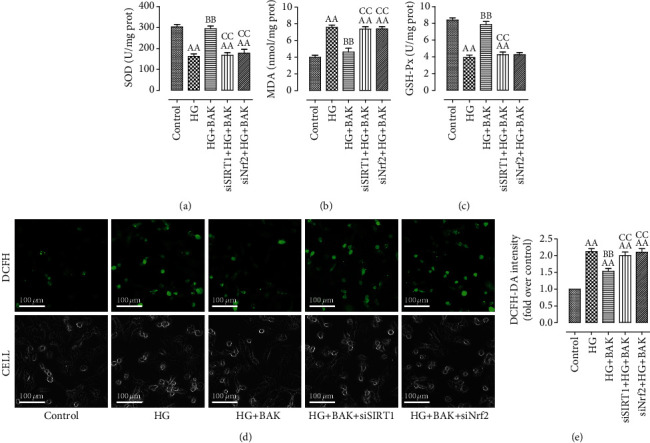
SIRT1 siRNA and Nrf2 siRNA transfection inhibited BAK-induced suppression on oxidative stress in HG-treated H9c2 cells. (a) Intracellular SOD activity. (b) Intracellular MDA content. (c) Intracellular GSH-Px activity. (d) Representative images of DCFH-DA staining (scale bar = 100 *μ*m). (e) DCFH-DA intensity. Data are presented as the mean ± SEM (*n* = 6 in each group). ^aa^*P* < 0.01 vs. the control group, ^bb^*P* < 0.01 vs. the HG group, and ^cc^*P* < 0.01 vs. the HG+BAK group.

In accordance with our *in vivo* results, BAK also improved SOD and GSH-Px activities and reduced MDA content in HG-treated H9c2 cells, whereas SIRT1 siRNA and Nrf2 siRNA abolished these effects (Figures 11(a)–11(c)). In addition, DCFH-DA fluorescence, used to detect intracellular ROS generation, has shown that BAK treatment significantly inhibited HG-triggered ROS production in the HG+BAK group. Consistently, SIRT1 siRNA and Nrf2 siRNA transfection blunted this antioxidative effect (Figures 11(d) and 11(e)).

Taken together, these data indicated that BAK could attenuate cellular death and oxidative damage in HG-cultured H9c2 cells via SIRT1 and Nrf2 signaling.

### 3.9. Nrf2 Acted as a Downstream Target of SIRT1 in Mediating the Cardioprotective Effects of BAK

And lastly, we investigated the regulatory relationship between SIRT1 and Nrf2. Results from both *in vivo* and *in vitro* research showed that EX527 or SIRT1 siRNA not only markedly suppressed BAK-induced promotion in expression and activity of SIRT1 but also abolished BAK-induced nuclear accumulation of Nrf2 (Figures 1 and 9(c)–9(f)). In the meantime, notwithstanding the remarkably inhibition of BAK-induced nuclear localization of Nrf2 by Nrf2 siRNA, it had little effect on SIRT1 expression or activity (Figures 9(c)–9(f)). In summary, these data indicated that Nrf2 might function downstream of SIRT1 signaling in mediating the cardioprotective actions of BAK against diabetic cardiomyopathy.

## 4. Discussion

The present study revealed that in the setting of diabetic cardiomyopathy, BAK reversed the adverse effects of hyperglycemia-induced oxidative stress in diabetic hearts, as demonstrated by improvement of cardiac function, mitigation of pathological cardiac hypertrophy, prevention of myocardial fibrosis, and elevation of cardiomyocyte survival rate. Mechanistically, the above protective capabilities of BAK were in part mediated by SIRT1/Nrf2-related antioxidative activities, including increased production of antioxidants, decreased generation of ROS, and suppressed expressions of fibrosis- and hypertrophy-associated molecules. So far as we know, it is the first research concerning the cardioprotective effects and potential underlying mechanisms of BAK against diabetic cardiomyopathy.

The incidence of DM is rising at an alarming rate, making it one of the most prevalent disorders that threaten the world health [37]. Clinical evidence obtained from population-based observational studies has indicated that structural and functional abnormalities in diabetic hearts adversely affect the prognosis of diabetic patients [38, 39]. These cardiac pathophysiological changes, induced by the metabolic alterations in DM, were defined as a distinct clinical entity named diabetic cardiomyopathy, which is initially characterized by myocardial fibrosis and cardiac remodeling, later by diastolic and systolic dysfunctions, and eventually by clinical heart failure [5]. As yet, due to the severe consequences of diabetes-associated hyperglycemia, diabetic cardiomyopathy has become a leading cause responsible for the greater risk of morbidity and mortality in diabetic patients [34]. Considering that conventional antidiabetic drugs cannot completely prevent the occurrence and progression of this cardiovascular complication [40, 41], searching for a novel agent targeting the pathophysiology and molecular mechanisms involved in diabetic cardiomyopathy could be a promising candidate therapy.

Bakuchiol (BAK), a bioactive natural meroterpene isolated from the seeds of *Psoralea corylifolia*, has long been used as a traditional Chinese medicine [11]. Crucially, previous studies have observed the favorable effects of BAK in the setting of diabetes. Seo et al. noted that BAK significantly reduces blood glucose levels, improves glucose tolerance, and increases serum insulin levels in streptozotocin-induced diabetic mice [17]. Krenisky et al. confirmed that the oral administration of BAK reduces blood glucose levels in db/db mice with type 2 diabetes [18]. Moreover, growing attention has also been paid to its protective actions on cardiovascular performance. Wang et al. demonstrated that BAK protects against pathological cardiac hypertrophy by blocking the NF-*κ*B signaling pathway [42]. Feng et al. reported that BAK attenuates myocardial ischemia reperfusion injury by maintaining mitochondrial function [15]. Additionally, Kassahun et al. found that BAK induces vasodilation in rat arteries through both endothelium-dependent and endothelium-independent mechanisms [43]. However, the relationship between BAK and diabetic cardiomyopathy has not been clarified thus far. In this study, we first observed that BAK treatment could significantly increase cell viability and prevent cell apoptosis in HG-cultured H9c2 cells. Then, the *in vivo* study further verified that BAK supplementation not only blocked myocardial hypertrophic response and alterations in left ventricular mass but also attenuated interstitial and perivascular fibrosis and collagen deposition. And the improvement of above structural abnormalities ultimately alleviated the cardiac dysfunction in the BAK-treated mice. Therefore, the administration of BAK may be a potential approach for the intervention of diabetic cardiomyopathy.

Evidence has shown that the oxidative stress in human diabetic hearts was overwhelming, which has been proven to be one of the pivotal roles in the development and progression of diabetic cardiomyopathy [5, 7, 44]. The metabolic milieu associated with DM, such as hyperglycemia, hyperlipidemia, and hyperinsulinemia, unduly increased ROS generation from mitochondrial and extramitochondrial sources [7, 45]. And the overly stimulated ROS production further triggered the oxidative stress in the diabetic myocardium, accompanied by immoderately exhausted endogenous antioxidants and excessively accumulated prooxidative damage products [6, 46]. At the same time, continuously boosted state of oxidative stress altered multiple molecular pathways within the cardiomyocytes, finally leading to the myocyte apoptosis or necrosis [9, 47]. More importantly, the pathogenies of diabetes-associated cardiac fibrosis and hypertrophy were proven to be closely connected with increased oxidative stress. On the one hand, overproduction of ROS could induce TGF-*β1*/Smad3 signaling activation, which promoted the expressions of several fibrotic markers, causing the proliferation of cardiac fibroblasts and collagen synthesis [48, 49]. On the other hand, increased oxidative stress could lead to the switch in the hypertrophy-related gene MHC expression from the *α*-MHC isoform to the *β*-MHC isoform, which marked the progression of cardiac hypertrophy [7, 50]. Hence, to inhibit ROS generation and relive the oxidative stress will be a very promising treatment strategy for diabetic cardiomyopathy. Intriguingly, numerous studies have provided supports for the use of BAK as an effective antioxidant. Haraguchi et al. found that BAK showed broad antioxidative activities in rat liver microsomes and mitochondria [51]. Adhikari et al. revealed the importance of the terpenoid moiety of BAK in controlling its antioxidant action via radical scavenging [52]. Moreover, thanks to its highly efficient antioxidative capacity, BAK has been certified to exhibit protective roles against multiple diseases in various organs or tissues [13–16], which greatly prompts us to confirm this effect in diabetic cardiomyopathy. As expected, data collected in our study showed that BAK treatment markedly improved SOD and GSH-Px activities and reduced MDA content, as well as inhibited hyperglycemia-triggered ROS production, thus alleviating myocardial oxidative stress both *in vivo* and *in vitro*. Furthermore, from the fibrosis-related protein and hypertrophy-related gene levels, BAK administration remarkably inhibited the expression of *α*-SMA and suppressed the activation of TGF-*β1*/Smad3 signaling. Meanwhile, mRNA levels of ANP and BNP were significantly decreased, and gene MHC expression underwent canonical changes from *β*-MHC to *α*-MHC.

To further unravel the molecular mechanisms implicated in the antioxidative effects of BAK against diabetic cardiomyopathy, we have focused on the SIRT1/Nrf2 signaling pathway. SIRT1, a protein widely expressed in mammalian cells, is highly sensitive to the cellular redox states and has recently been intensively investigated in the cardiovascular system [53]. Immense amounts of concrete research have demonstrated that endogenous SIRT1 is tightly linked to the cardioprotection of various cardiovascular diseases for its pivotal role in increasing resistance to oxidative damage [19, 54]. As for Nrf2, another vital antioxidant sensor, it confers maintenance of cellular defense system by promoting the expressions of antioxidant molecules when its position shifts from the cytoplasm to the nucleus where it binds to AREs [55, 56]. Many previous studies have already revealed the two redox-sensitive proteins closely implicated in the therapeutic effects on diabetic cardiomyopathy [21, 46, 57, 58]. More importantly, SIRT1 is involved in the regulation and control of oxidative stress that are associated with diverse stimulus, which appears to be exerted by activating Nrf2, indicating that Nrf2 serves as an important downstream target of SIRT1 signaling [25, 26, 31, 59]. Additionally, novel observations have suggested that BAK inhibits ROS generation in cardiomyocytes via SIRT1. And Shoji et al. found that BAK is a phenolic isoprenoid with novel enantiomer-selective anti-influenza A virus activity involving Nrf2 activation [60]. Therefore, the above findings strongly intrigued us to test the connection between the SIRT1/Nrf2 signaling and BAK protective effects in this diabetic complication. Within expectation, our results showed that BAK treatment reversed the downward trends of SIRT1 expression and Nrf2 nuclear translocation caused by hyperglycemia, while EX527, SIRT1 siRNA, or Nrf2 siRNA abolished this effect, finally nullifying the antioxidative actions of BAK. In the meantime, EX527 and SIRT1 siRNA not only blocked the BAK-induced increase in SIRT1 expression and activity but also inhibited the Nrf2 nuclear accumulation, but Nrf2 siRNA had little effect on SIRT1 expression or activity, suggesting that SIRT1 functioned upstream of Nrf2 signaling. Consequently, we concluded that BAK ameliorated diabetic cardiomyopathy by reducing myocardial oxidative damage in a SIRT1/Nrf2 signaling-dependent pathway.

In conclusion, we have demonstrated for the first time that BAK treatment can effectively retard the progression of pathological cardiac hypertrophy and inhibit the development of cardiac fibrosis against diabetic cardiomyopathy, which ultimately alleviate the impairment of the cardiac function. These effects were primarily ascribed to the activation of SIRT1/Nrf2 signaling and its followed attenuation of myocardial oxidative stress, as SIRT1/Nrf2 signaling played an important role in elevating the antioxidant production and reducing the ROS generation in diabetic myocardium. Our study strongly suggested that long-term BAK supplement may be chosen as a therapeutic intervention for treating diabetics with cardiac abnormalities. In the future, experiments concerning other upstream and (or) downstream signals of SIRT1 should be warranted to establish a more comprehensive molecular mechanisms of BAK protective actions, and additional trials using larger animal models must be operated before enabling its application towards clinical practice.

## Figures and Tables

**Table 1:** Transfection siRNAs used in the present study.

siRNA	Sense	Antisense
SIRT1	5′-CCA GUA GCA CUA AUU CCA ATT-3′	5′-UUG GAA UUA GUG CCA CUG GTT-3′
Nrf2	5′-GAG GAU GGG AAA CCU UAC UTT-3′	5′-AGU AAG GUU UCC CAU CCU CTT-3′
Negative control	5′-UUC UCC GAA CGU GUC ACG UTT-3′	5′-ACG UGA CAC GUU CGG AGA ATT-3′

SIRT1: silent information regulator 1; Nrf2: nuclear factor-erythroid 2-related factor 2.

**Table 2:** Real-time quantitative RT-PCR primers used in the present study.

Gene	Forward	Reverse
ANP	5′-ACCTGCTAGACCACCTGGAG-3′	5′-CCTTGGCTGTTATCTTCGGTACCGG-3′
BNP	5′-GAGGTCACTCCTATCCTCTGG-3′	5′-GCCATTTCCTCCGACTTTTCTC-3′
*α*-MHC	5′-TGCACTACGGAAACATGAAGTT-3′	5′-CGATGGAATAGTACACTTGCTGT-3′
*β*-MHC	5′-ACTGTCAACACTAAGAGGGTCA-3′	5′-TTGGATGATTTGATCTTCCAGGG-3′
GAPDH	5′-AGAACATCATCCCTGCATCC-3′	5′-AGTTGCTGTTGAAGTCGC-3′

ANP: atrial natriuretic polypeptide; BNP: brain natriuretic peptide; *α*-MHC: *α*-myosin heavy chain; *β*-MHC: *β*-myosin heavy chain; GAPDH: glyceraldehyde-3-phosphate dehydrogenase.

## Data Availability

The data used to support the findings of this study are available from the corresponding author upon request.

## Supplementary Materials

Supplementary Materials Figure S1: the effects of BAK on the expression and activity of SIRT1 and nuclear accumulation of Nrf2 in normal hearts. (a) Representative blots. (b) SIRT1 expression. (c) Relative SIRT1 activity. (d) Nrf2 nuclear translocation. Data are presented as the mean ± SEM (*n* = 6 in each group). Figure S2: the effects of BAK on intracellular SIRT1 expression and activity and Nrf2 nuclear accumulation in normal H9c2 cells. (a) Representative blots. (b) SIRT1 expression. (c) Relative SIRT1 activity. (d) Nrf2 nuclear translocation. Data are presented as the mean ± SEM (*n* = 6 in each group).
