# Supplementary material for: Bakuchiol Alleviates Hyperglycemia-Induced Diabetic Cardiomyopathy by Reducing Myocardial Oxidative Stress via Activating the SIRT1/Nrf2 Signaling Pathway
Source: Oxid Med Cell Longev. 2020 Sep 30;2020:3732718. doi: 10.1155/2020/3732718 (PMC7545423; doi:10.1155/2020/3732718)
Supplement: Supplementary Materials — Figure S1: the effects of BAK on the expression and activity of SIRT1 and nuclear accumulation of Nrf2 in normal hearts. (a) Representative blots. (b) SIRT1 expression. (c) Relative SIRT1 activity. (d) Nrf2 nuclear translocation. Data are presented as the mean ± SEM (n = 6 in each group). Figure S2: the effects of BAK on intracellular SIRT1 expression and activity and Nrf2 nuclear accumulation in normal H9c2 cells. (a) Representative blots. (b) SIRT1 expression. (c) Relative SIRT1 activity. (d) Nrf2 nuclear translocation. Data are presented as the mean ± SEM (n = 6 in each group). [file 3732718.f1.docx]

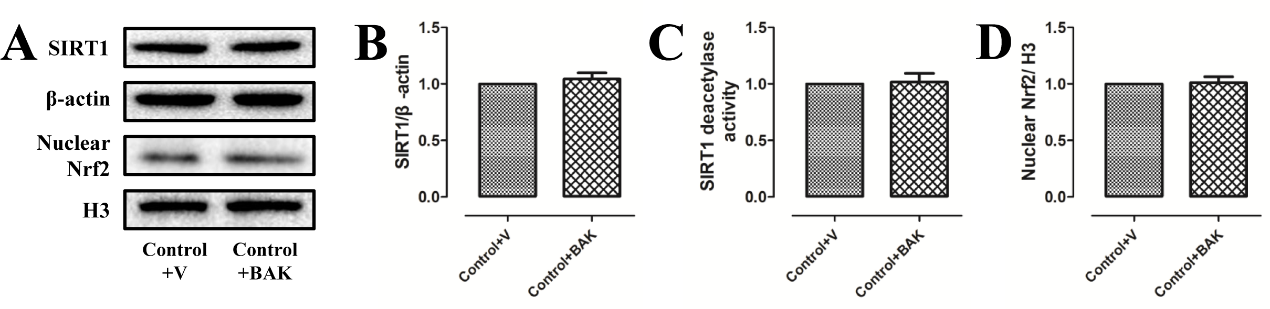


Figure S1: The effects of BAK on the expression and activity of SIRT1 and nuclear accumulation of Nrf2 in normal hearts. (a) Representative blots. (b) SIRT1 expression. (c) Relative SIRT1 activity. (d) Nrf2 nuclear translocation. Data are presented as the mean ± SEM. (n=6 in each group).


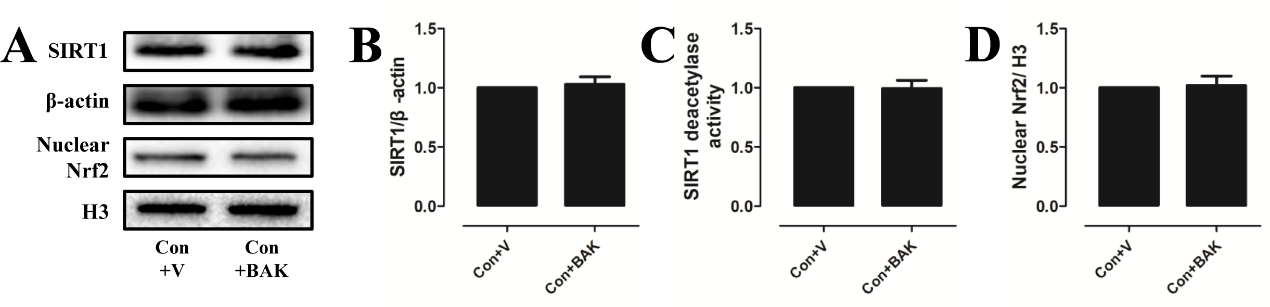


Figure S2: The effects of BAK on intracellular SIRT1 expression and activity and Nrf2 nuclear accumulation in normal H9c2 cells. (a) Representative blots. (b) SIRT1 expression. (c) Relative SIRT1 activity. (d) Nrf2 nuclear translocation. Data are presented as the mean ± SEM. (n=6 in each group).
